# Consequence of the magnocellular dysfunction on processing facial affect recognition in Schizophrenia

**DOI:** 10.1192/j.eurpsy.2022.411

**Published:** 2022-09-01

**Authors:** C. Marosi, Z. Fodor, G. Csukly

**Affiliations:** Semmelweis University, Department Of Psychiatry And Psychotherapy, Budapest, Hungary

**Keywords:** schizophrénia, Emotion recognition, theta ERSP, Perception

## Abstract

**Introduction:**

Magnocellular deficit in visual perception and impaired emotion recognition are core features of schizophrenia, however their relationship and the neurobiological underpinnings are still unclear.

**Objectives:**

The aim of our research was to investigate the oscillatory background of perception and emotion recognition in schizophrenia and to examine the relationship between these processes.

**Methods:**

Thirty-nine subjects with schizophrenia and forty healthy controls subjects were enrolled in the study; the two study groups did not differ in age, gender and education. In the visual paradigm the participants viewed magnocellular biased low-spatial frequency (LSF) and parvocellular biased high-spatial frequency (HSF) Gabor-patches and in the second paradigm happy, sad and neutral faces were presented, while 128-channel EEG was recorded.

**Results:**

Significantly weaker theta (4-7 Hz) event related synchronisation (ERS) was observed in patients compared to controls in the LSF condition, whereas in the HSF condition there was no difference between the two groups. Event related changes in theta amplitude were also found to be significantly weaker in patients compared to healthy controls in the emotion recognition task, which difference was disappeared after correction for ERS to LSF condition. In the correlational analysis theta activity in the magnocellular biased stimuli correlated significantly with theta activity in the emotion recognition task, while theta to parvocellular biased stimuli showed no similar correlation with emotion recognition.

**Conclusions:**

In schizophrenia, emotion recognition impairments are closely related to the dysfunction of the magnocellular system, which supports the bottom-up model of schizophrenia.

**Disclosure:**

No significant relationships.

